# An Approach to Function Annotation for Proteins of Unknown Function (PUFs) in the Transcriptome of Indian Mulberry

**DOI:** 10.1371/journal.pone.0151323

**Published:** 2016-03-16

**Authors:** K. H. Dhanyalakshmi, Mahantesha B. N. Naika, R. S. Sajeevan, Oommen K. Mathew, K. Mohamed Shafi, Ramanathan Sowdhamini, Karaba N. Nataraja

**Affiliations:** 1 Department of Crop Physiology, University of Agricultural Sciences, GKVK, Bengaluru, 560065, India; 2 National Centre for Biological Sciences, TIFR, GKVK campus, Bengaluru, 560065, India; Jawaharlal Nehru University, INDIA

## Abstract

The modern sequencing technologies are generating large volumes of information at the transcriptome and genome level. Translation of this information into a biological meaning is far behind the race due to which a significant portion of proteins discovered remain as proteins of unknown function (PUFs). Attempts to uncover the functional significance of PUFs are limited due to lack of easy and high throughput functional annotation tools. Here, we report an approach to assign putative functions to PUFs, identified in the transcriptome of mulberry, a perennial tree commonly cultivated as host of silkworm. We utilized the mulberry PUFs generated from leaf tissues exposed to drought stress at whole plant level. A sequence and structure based computational analysis predicted the probable function of the PUFs. For rapid and easy annotation of PUFs, we developed an automated pipeline by integrating diverse bioinformatics tools, designated as PUFs Annotation Server (PUFAS), which also provides a web service API (Application Programming Interface) for a large-scale analysis up to a genome. The expression analysis of three selected PUFs annotated by the pipeline revealed abiotic stress responsiveness of the genes, and hence their potential role in stress acclimation pathways. The automated pipeline developed here could be extended to assign functions to PUFs from any organism in general. PUFAS web server is available at http://caps.ncbs.res.in/pufas/ and the web service is accessible at http://capservices.ncbs.res.in/help/pufas.

## Introduction

Advances in sequencing technologies have opened up unlimited opportunities for better understanding of the molecular events occurring spatially and temporally during the growth and development of an organism. Large volumes of genomic and transcriptomic information have been developed and a broad spectrum of bioinformatic tools as well as experimental strategies have been adopted for their annotation. However, linking such huge gene sequence information with a biological meaning remains a challenge, leaving behind a major portion of the identified proteins as proteins of unknown function (PUFs) in public databases. About 16 and 30% of proteins are unannotated in bacteria and yeast genomes [1][2]. In eukaryotes, over 40% of the proteins encoded by genomes is reported to lack functional annotation [3][4]. In a model plant system *Arabidopsis thaliana*, approximately 30–34% of the total genome is composed of PUFs [5]. Several attempts have been reported in diverse organisms, to uncover the biological role of PUFs, enumerating their functional significance in growth, development, survival and response to adverse environmental conditions [6][7][8]. There is a need to assign function to PUFs for prospecting interesting genes until then, our understanding on the complexities in the growth and development of an organism and its interaction with the biotic and abiotic environment remains unclear. The functional annotation of all the PUFs based on laboratory experiments would be time consuming and expensive. Hence, several bioinformatic tools focusing on sequence similarity, co-expression, interactions, protein structures etc., have been widely used [9][3][10][11]. However, as evident from the prominent existence of PUFs in the genomes of many organisms, high throughput pipelines and methodologies to rapidly annotate PUFs and to elucidate their biological roles would be useful. In this study, we are reporting a strategy to enumerate the functions of the PUFs generated through any sequencing platforms.

We attempted to annotate the PUFs identified from expressed sequence tag (EST) library of mulberry leaf tissue exposed to drought stress. The preliminary functional annotation of the library yielded diverse proteins of known functions (PKFs), where as several genes have been identified as PUFs. For analysis of PUFs, we developed a pipeline using freely available bio-informatics tools and attempted to assign putative functions to many mulberry PUFs. Further, for rapid and high-throughput annotation of PUFs, we developed an automated pipeline and tested its application. We also examined the relevance of three annotated PUFs by *in-vivo* gene expression in mulberry. The stress-responsive PUFs identified in this study could be subjected for further functional characterization to elucidate their significance in plant growth and development, as well as in abiotic stress tolerance. This approach for the annotation of PUFs would be useful in assigning functions to many uncharacterized proteins identified from diverse transcriptome and genome datasets, irrespective of species of the organism and their growth, development and environmental conditions.

## Materials and Methods

***Ethics statement*:** In this study, we used mulberry (*Morus alba* L.) genotype, Dudia white, which is being maintained in the Department of Crop Physiology, University of Agricultural Sciences, GKVK, Bengaluru, India. There is no need of formal approval for this type of study, as the research is carried out in a public sector (non-profit) organization, and the study does not involve any genetic modification of the genotype used.

### Plant material, RNA extraction, cDNA library preparation and sequencing

Stem cuttings of mulberry (*Morus alba* L.) genotype, Dudia white were used to generate healthy plants in pots (30kg capacity) filled with potting mixture. The plants were grown in the garden of the Department of Crop Physiology, University of Agricultural Sciences, GKVK, Bengaluru, India. Two months old healthy plants were subjected to different levels of drought stress (70–80% mild, 55–65% moderate and 40–50% severe) at soil field capacity (FC), imposed by gravimetric approach [12].

Leaf tissue was collected from the plants experiencing different levels of drought stress and total RNA was isolated according to a modified protocol [13]. From the total RNA, messenger RNA (mRNA) was extracted using mRNA isolation kit (Oligotex mRNA Mini kit Qiagen, CA, USA) and equal amounts of mRNA were pooled. The mRNA enriched fraction was converted to 454 barcoded cDNA library as reported [14]. In brief, from mRNA (200ng), cDNA was synthesized using cDNA synthesis kit (SuperScript Double-Stranded cDNA Synthesis Kit, Invitrogen, CA, USA) with 100mM random hexamer primers (Fermentas, USA). The double-stranded cDNA synthesized was purified and nebulized using kit supplied with the GS Titanium Library Preparation kit (454 Life Sciences, CT, USA) following their recommendations (30psi for 1minute) and purified with a QIAquick PCR minelute column (Qiagen, CA, USA) and eluted in 50μL elution buffer (EB). The sample library prepared was analysed using a Qubit fluorometer (Invitrogen, CA) and average fragment sizes were determined on the Bioanalyzer (Agilent, CA, USA). The process of library preparation, emulsion-based clonal amplification and sequencing on the 454 Genome Sequencer FLX Titanium system were performed according to the manufacturer’s instructions (454 Life Sciences, CT, USA; M/s. Sasya Gentech, Bangalore, India). Signal processing and base calling were performed using bundled 454 data analysis software v2.6.

### *De novo* assembly and annotation

**Fig 1:**
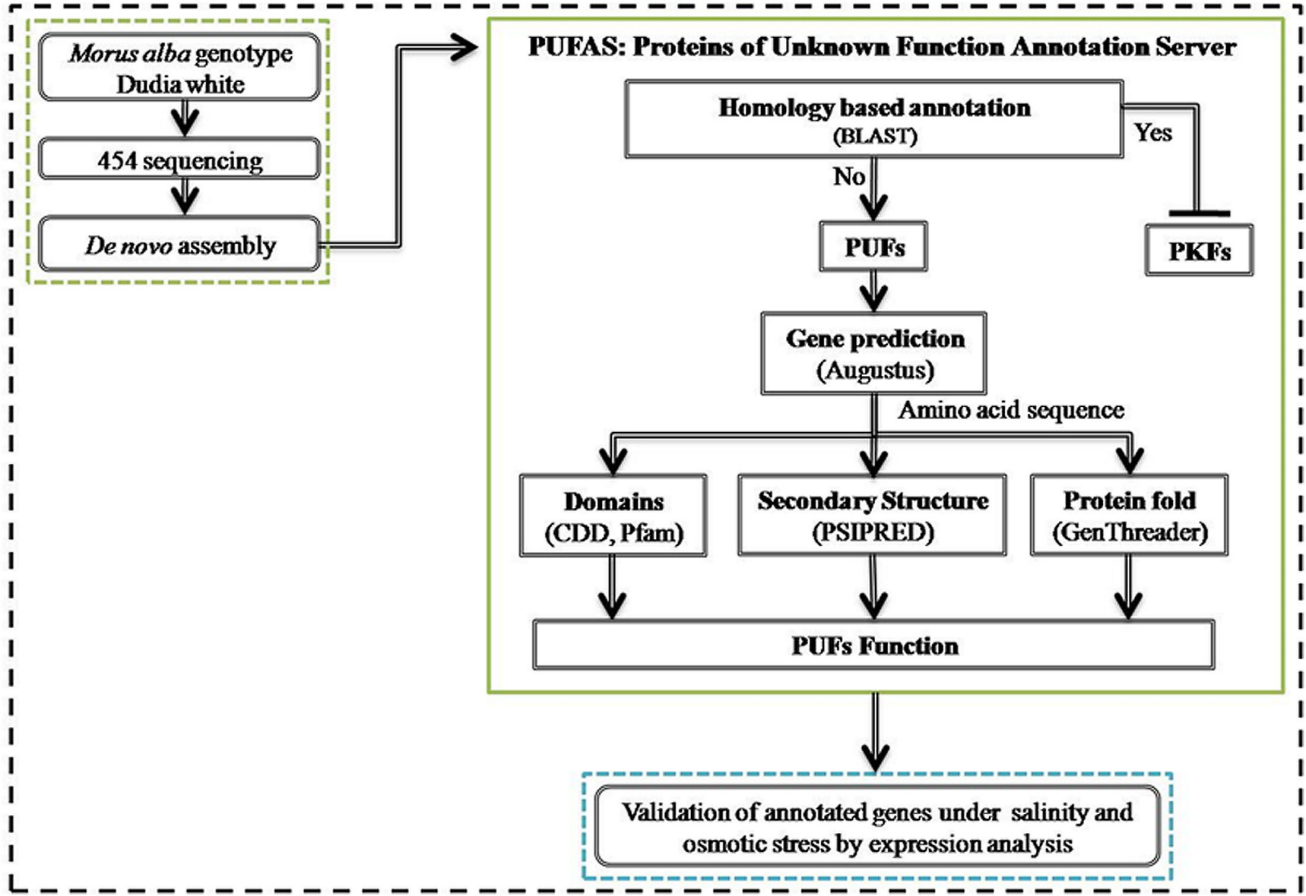
The schematic representation of different events staring from library generation by 454 sequencing, assembly, annotation and other analysis performed.

The DNA sequences obtained were processed and contigs were assembled using *de novo* Roche 454’s Newbler from a non-normalized mulberry cDNA library [14]. The transcriptome data was submitted to the National Center for Biotechnology Information’s (NCBI) Sequence Read Archive (SRA) with the study accession number of SRP047446. The contigs were annotated using blastx against NCBI-nr (http://blast.ncbi.nlm.nih.gov/Blast.cgi) and broadly classified as PKFs and PUFs. The PUFs were selected for function prediction by bioinformatic approaches and, the randomly selected PUFs were used for experimental validation. The schematic representation of the events followed to process the ESTs is depicted in Fig 1.

### Gene prediction from contigs

The gene prediction was carried out using available online tools like Softberry’s HMM-based *ab-initio* gene structure prediction by FGENESH [15] with *Populus trichocarpa* as reference genome and AUGUSTUS (*A*. *thaliana*), a HMM-based eukaryotic gene prediction server [16] and the longest gene length prediction was accepted as the gene model.

### Computational annotation of PUFs

Searches were performed in Pfam database [17] and Conserved Domain Database (CDD) of the NCBI [18] for annotating the PUFs for the presence of domains and protein family in the targeted protein sequences. Fold prediction tools, like GenTHREADER [19], PHYRE2 [20] and 3DPSSM [21] were used to predict the compatible folds and associate to the function.

### Development of Server for Function Annotation of Proteins of Unknown Function (PUFAS)

PUFAS web interface is developed using Javascript, HTML and CSS. Input for PUFAS is contigs, from that it predicts the possible function of an input sequence. In PUFAS, analysis was performed using the NCBI blastx for finding the homologous. Based on the preliminary annotation, the PUFs were further taken for gene prediction using AUGUSTUS, and from this the amino acid sequence was used as an input for tools like Pfam and CDD for identification of domains associated with query sequence. The GenThreader was used for the fold prediction and all these tools implemented with options to choose user-defined statistics values. Function could be assigned on the basis of the predicted domains and fold. User can download the output as a batch file.

### Phylogenetic analysis

To reveal the divergence of one of the unknown genes, PUF39, in other plant genomes, a BLAST search was performed to identify the homologous genes (blastp with default parameters) against the NCBI-nr database. All hits below an E-value of 0.001 were retrieved as homologous sequences from other genomes in the GenBank database. Multiple sequence alignment was performed using ClustalW, and the alignment edited manually, tree was constructed using neighbour-joining (NJ) method in MEGA5.0 at a bootstrap value of 1000 [22].

### Protein-protein interactions

Selected PUFs were queried for protein-protein interactions using the STRING database by applying a conservative score threshold of 0.7 [23].

### Expression analysis of selected PUFs

#### Stress treatments

To study the expression pattern of the selected PUFs under other abiotic stresses, experiments were conducted under controlled laboratory conditions. Salinity and oxidative stresses were simulated by exposing the freshly collected intact twigs of mulberry to 250mM sodium chloride (NaCl) and 15μM methyl viologen (MV), respectively. Leaf tissues were collected at 6, 12, 24 and 48 hours after the stress imposition, while water treated twigs served as control.

#### Quantitative Real-Time PCR (qRT-PCR)

The total RNA was isolated from 100mg of the leaf tissue collected from the respective stress treatments according to a modified protocol [13]. About 3μg of total RNA was reverse transcribed to synthesize first strand cDNA using the RevertAid First Strand cDNA Synthesis Kit (Thermo Scientific, USA). The cDNA was used as the template for expression analysis and the house keeping gene, elongation factor (*elf*) was used as the internal control. The qRT-PCR was performed in a q-PCR machine (Opticon2, MJ Research, USA), with the fluorescent dye SYBR-green (SYBR Premix Ex Taq, Perfect Real Time, Takara, Japan) under standardized PCR conditions using target specific primers as listed (S1 File). The relative transcript level was calculated from three independent replications; calculated using comparative ΔCt method [24] and student t-test was performed (p = 0.05).

## Results

### Sequencing and annotation of the drought specific transcriptome of mulberry

**Fig 2:**
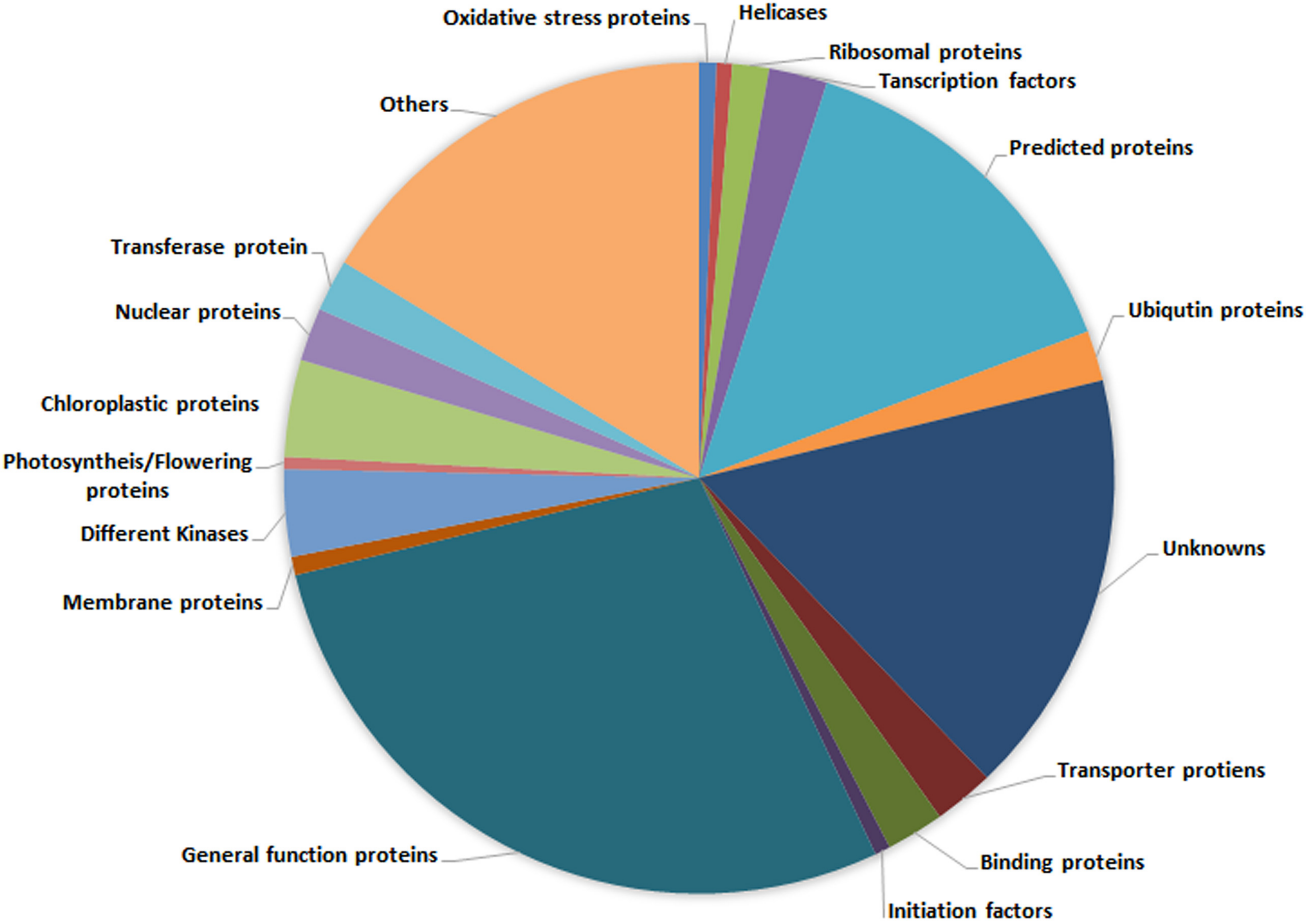
Distribution of selected GO terms based on NCBI-BLAST analysis of mulberry transcriptome generated from drought stressed leaf tissue.

The cDNA library developed from drought stressed leaf tissues of mulberry yielded 10,190 ESTs. As a preliminary stage of library analysis, all the ESTs were searched at NCBI against nr database with a stringent E-value of 1e^-5^, from which 5319 ESTs were annotated and classified into PKFs and PUFs. The PKFs belong to various functional as well as regulatory proteins such as kinases, ribosomal proteins, membrane proteins, transporters, transcription factors (TFs), etc. (Fig 2). Detailed GO annotation information is provided as S2 File. The remaining ESTs were annotated as uncharacterized, hypothetical and unknown proteins which we considered as PUFs, as they lacked an experimental backup for function prediction. In our study, which was initiated in January 2014, we considered some of the PUFs, which were above 500bp, for functional annotation.

### Gene prediction from contigs

In order to confirm the gene boundaries, we employed FGENESH and AUGUSTUS, the prediction which gave longest open reading frame (ORF) were selected for further annotation and experimental study (S3 File).

### Annotation of PUFs using computational tools

Sequence and structure based computational tools were utilized for the annotation of PUFs. Pfam and CDD search could identify the domains and families present in selected PUFs. The sequence analyses and domain association for all PUFs considered in the study are presented (S3 File). Following this, PUFs were also subjected to structure based functional analysis tools (S3 File). Integrating the sequence and structure based analysis, gene functions were predicted and data generated on some of the PUFs are presented in Table 1. For high throughput and rapid annotation of PUFs, we developed a web server PUFAS and implemented a RESTFul web service API for the programmatic access to the tool that can enable large-scale annotation of PUFs from a genome.

**Table 1:** Annotated PUFs using the computational tools.

Name of the contig	Annotation from NCBI	Predicted function
contig00286	Unknown	Thioredoxin like protein
contig00529	Unknown	ACT domain containing protein
contig01194	Unknown	Universal stress protein like
contig01391	Unknown	Ankyrin repeat protein
contig01796	Unknown	Transferase like
contig01963	Unknown	CBS domain containing protein
contig02017	Unknown	GroEL-like chaperone
contig02101	Unknown	Oxidoreductase like
contig02328	Unknown	RNA binding protein
contig02670	Unknown	Aldo-keto reductase like
contig04385	Unknown	Major intrinsic protein
contig04437	Unknown	Transketolase like
contig04699	Unknown	GDP-fucose protein O-fucosyltransferase
contig04820	Unknown	Aldehyde reductase
contig04823	Unknown	GDP-fucose protein O-fucosyltransferase
contig04921	Unknown	Ankyrin repeat protein
contig5180	Unknown	Dehydrogenase like
contig05224	Unknown	Protein phosphatase like
contig05320	Unknown	Dehydrogenase like
contig05347	Unknown	Ferritin like protein
contig05421	Unknown	Dehydrogensase/ reductase
contig05454	Unknown	Late embryogenesis abundant protein like
contig05496	Unknown	Acyl esterase like
contig05505	Unknown	Metallophosphatase
contig05537	Unknown	Ubiquitin like protein
contig05592	Unknown	Dehydrogenase like
contig05650	Unknown	ATP synthase
contig05740	Unknown	Transferase like
contig05864	Unknown	Hydrolase like
contig05991	Unknown	TPR protein
contig06030	Unknown	Sulphite exporter like
contig06333	Unknown	Chlorophyll a-b binding protein
contig06433	Unknown	Chlorophyll a-b binding protein
contig06595	Unknown	Trios phosphate isomerase
contig06596	Unknown	Rab5 like protein
contig06640	Unknown	Phosphoglucomutase
contig06735	Unknown	PLATZ like transcription factor
contig06750	Unknown	Elongation factor protein
contig06773	Unknown	Protein kinase like
contig06932	Unknown	RNA binding protein like
contig07145	Unknown	Rab like protein
contig07540	Unknown	Dormancy/ auxin associated protein
contig07570	Unknown	Ras related protein
contig07599	Unknown	Cytochrome c oxidase
contig07639	Unknown	Proteosome regulatory complex subunit like
contig08042	Unknown	Aldolase like
contig08184	Unknown	Ubiquitin conjugating enzyme like
contig08330	Unknown	Rab like protein
contig08474	Unknown	Ribosomal protein like
contig08640	Unknown	Hydrolase like
contig08772	Unknown	Metal transport protein
contig08856	Unknown	Pholem protein like
contig08939	Unknown	Ubiquinol-cytochrome c reductase complex subunit like
contig09315	Unknown	Dehalogenase like
contig09355	Unknown	Elongation factor like protein
contig09421	Unknown	Ribosomal protein like
contig00062	Hypothetical	Metallothionein like
contig00355	Hypothetical	Pepsin like
contig00754	Hypothetical	Ca binding epidermal growth factor like protein
contig01002	Hypothetical	Late embryogenesis abundunt protein like
contig01413	Hypothetical	Heavy metal binding protein like
contig01734	Hypothetical	Leucine rich repeat protein
contig01852	Hypothetical	Esterase like
contig01866	Hypothetical	KH RNA binding protein
contig01896	Hypothetical	Protein kinase like
contig01986	Hypothetical	Armadillo repeat protein
contig02310	Hypothetical	Perforin like
contig02336	Hypothetical	Ribosomal protein like
contig02443	Hypothetical	SKP1 like protein
contig02598	Hypothetical	Dehydrogense lke
contig02665	Hypothetical	Hydrolase like
contig02872	Hypothetical	Plant retinoblastoma associated protein
contig03421	Hypothetical	Transport protein like
contig03453	Hypothetical	Phosphoglucomutase
contig04442	Hypothetical	WD repeat protein
contig04444	Hypothetical	Acid protease like
contig04469	Hypothetical	SART 1 family protein
contig04481	Hypothetical	Tubulin associated protein
contig04536	Hypothetical	Myb like protein
contig04717	Hypothetical	Transporter like
contig04834	Hypothetical	Vacuolar sorting associated protein
contig04885	Hypothetical	PH domain containing protein
contig04956	Hypothetical	Transferase like
contig04977	Hypothetical	Phosphoprotein like
contig00200	Uncharacterized	DNA binding protein
contig00467	Uncharacterized	Late embryogenesis abundant protein like
contig00638	Uncharacterized	Transferase like
contig00917	Uncharacterized	Ribosomal protein like
contig01190	Uncharacterized	Zinc finger protein
contig01205	Uncharacterized	Transferase like
contig01222	Uncharacterized	Pseudouridine synthase like
contig01234	Uncharacterized	Transferase like
contig01406	Uncharacterized	Peroxidase like
contig01408	Uncharacterized	Peroxidase like
contig01644	Uncharacterized	Hydrolase like
contig01646	Uncharacterized	Transferase like
contig01671	Uncharacterized	RNA binding protein
contig01710	Uncharacterized	Hydrolase like
contig01711	Uncharacterized	Hydrolase like

**Fig 3:**
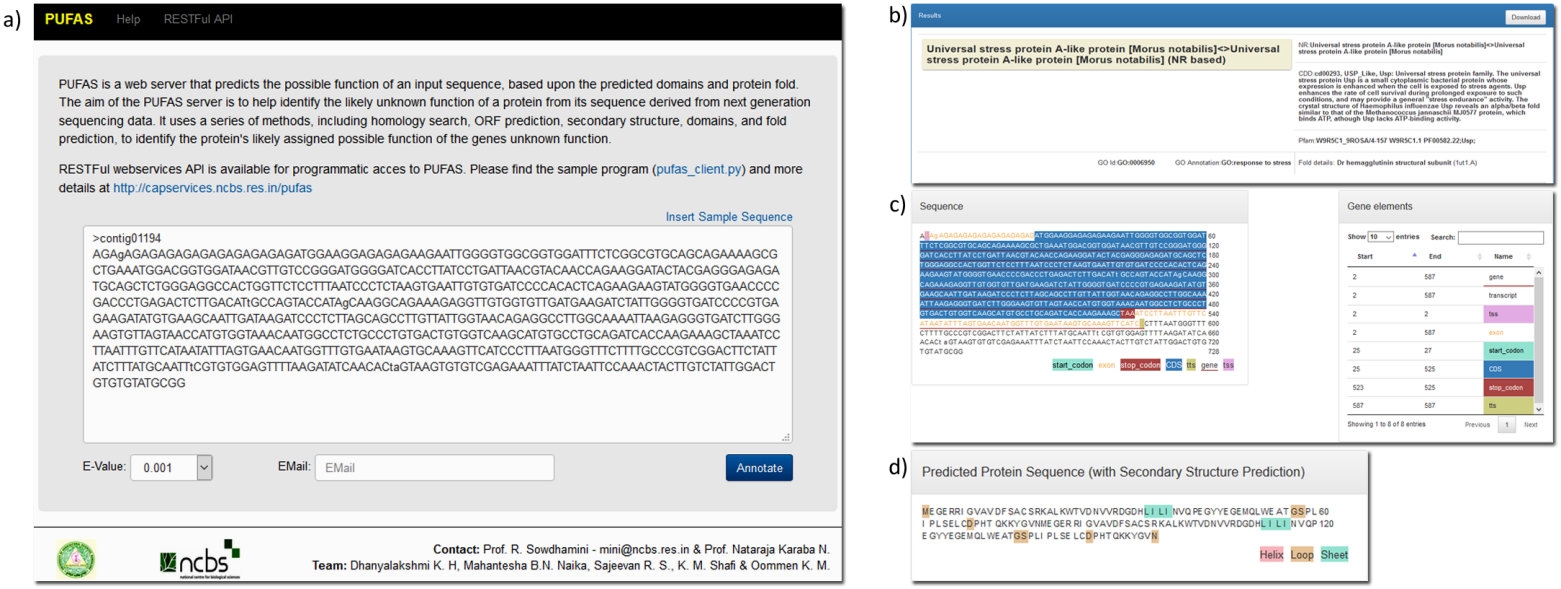
Web interface of PUFAS. a) Option to input of contigs identified from a genome by using next-generation sequencing, b) Output of Pfam and CDD, c) Gene identification and its elements, d) Amino acid sequence of a given contig.

PUFAS provides a web platform for performing PUFs analysis of next generation sequencing data. A user can submit a single or list of contigs from genomics or transcriptomics experiments, and can select statistical values to perform the annotation analyses. A successful PUFAS run provides batch file contains predicted gene boundaries, associated domains, secondary structure and fold predicted. The downloadable files can be used to filter significance level association of annotation based on user need. The automated server showed similar results as in case of manual annotation. This has been illustrated with the help of one of the PUFs from our study (Fig 3a–3d). The web service API enables the programmatic access of the tool, submit the sequence and save the result on the local desktop or laptop independent of the geographical location, the programming language or the computer platform. Sample python program to access the PUFAS web service is available in the website and as S4 File.

From the analysis, the result of three most promising PUFs, where domain association was strong, is presented in detail (Table 2). The stress-responsive nature of the selected genes was validated by qRT-PCR. As per the sequence and fold predictions, one of the genes, the PUF3 is predicted to have an adenine nucleotide-binding domain-like fold and belongs to the USP-like protein family. PUF39 is predicted to be a PLATZ1-like TF having a plant AT-rich binding region by sequence-based domain association studies while the structure based analysis could not derive an annotation, within acceptable limits of E-value. PUF42 was predicted to be an RNA-binding protein with a conserved RRM1 motif, but did not find a significant fold in the 3DPSSM and GenTHREADER. However, using PHYRE2, we could associate this protein to retain an RNA binding domain with coverage of 56% of the amino acid residues with 99.8% confidence. The salient results of these three PUFs are presented in Table 2.

**Table 2:** Sequence and structure based annotation of selected PUFs.

Contig ID	PUFs	Sequence based annotation		Structure based annotation			Annotation
		CDD	PFam	3DPSSM	PHYRE	GenTHREADER	
Contig 01194	PUF3	Universal stress protein family	Universal stress protein family	ETFP adenine nucleotide-binding domain-like	Adenine nucleotide alpha hydrolase-like	Adenine nucleotide alpha hydrolase-like	*MaUSP*-like
Contig 06735	PUF39	PLATZ1 transcription factor	PLATZ1 transcription factor	Immunoglobulin-like beta-sandwich	Gene regulation, hydrolase	DNA clamp	*MaPLATZ1*- like
Contig 06932	PUF42	RNA recognition motif	RNA recognition motif	No Prediction	RNA binding protein	Ferredoxin-like	*MaRRM1*- like

### Phylogenetic analysis

**Fig 4:**
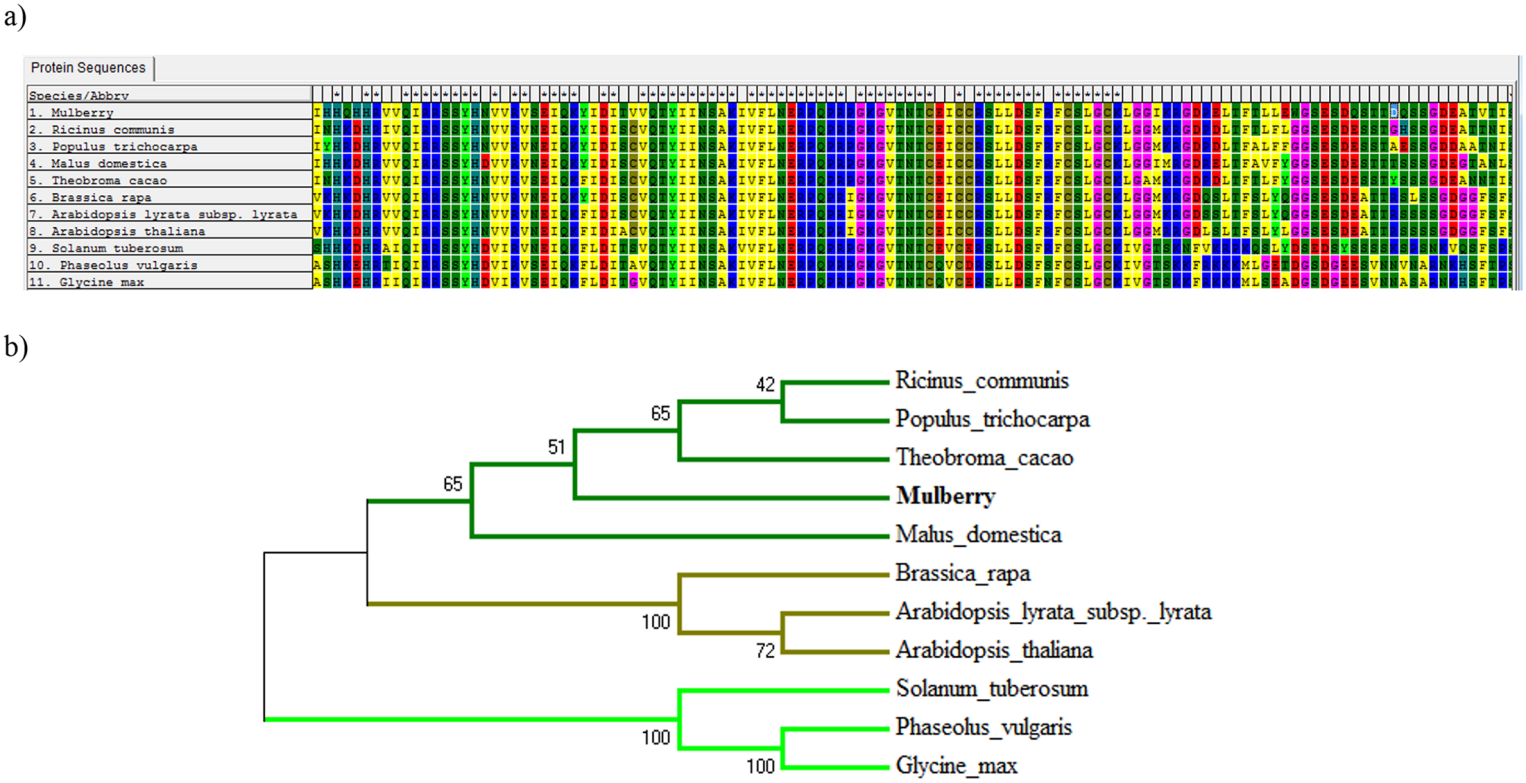
Phylogenetic analysis of selected PUF. a) Multiple sequence alignment of mulberry PUF39 with homologous genes from other plant genomes. b) Unrooted phylogenetic tree of PUF39. The Genbank accession numbers of the sequences are *Ricinus communis* (XP_002510750.1), *Populus trichocarpa* (XP_002301890.1), *Malus domestica* (XP_008381650.1), *Theobroma cacao* (XP_007018633.1), *Brassica rapa* (XP_009149581.1), *Arabidopsis lyrata* subsp. *Lyrata* (XP_002890421.1), *Arabidopsis thaliana* (NP_001117322.1), *Solanum tuberosum* (XP_006352862.1), *Phaseolus vulgaris* (XP_007131503.1) and *Glycine max* (XP_003540084.1).

Computational methods to analyze the phylogenetic relationships have been instrumental in annotation of protein functions [25] for which MEGA5.0 was used. We extracted protein sequence from the genomes having homologues to one of the mulberry PUFs i.e., PUF39 to understand the features of the similar genes and sequence conservation in other genomes (Fig 4a) and an unrooted phylogenetic tree was constructed (Fig 4b). The average sequence identity among species is 53% and the PUF39 was observed to cluster with other tree species; notably homologues from *Leguminace* and *Brassicacae* family members clustered together (Fig 4b).

### Expression analysis

**Fig 5:**
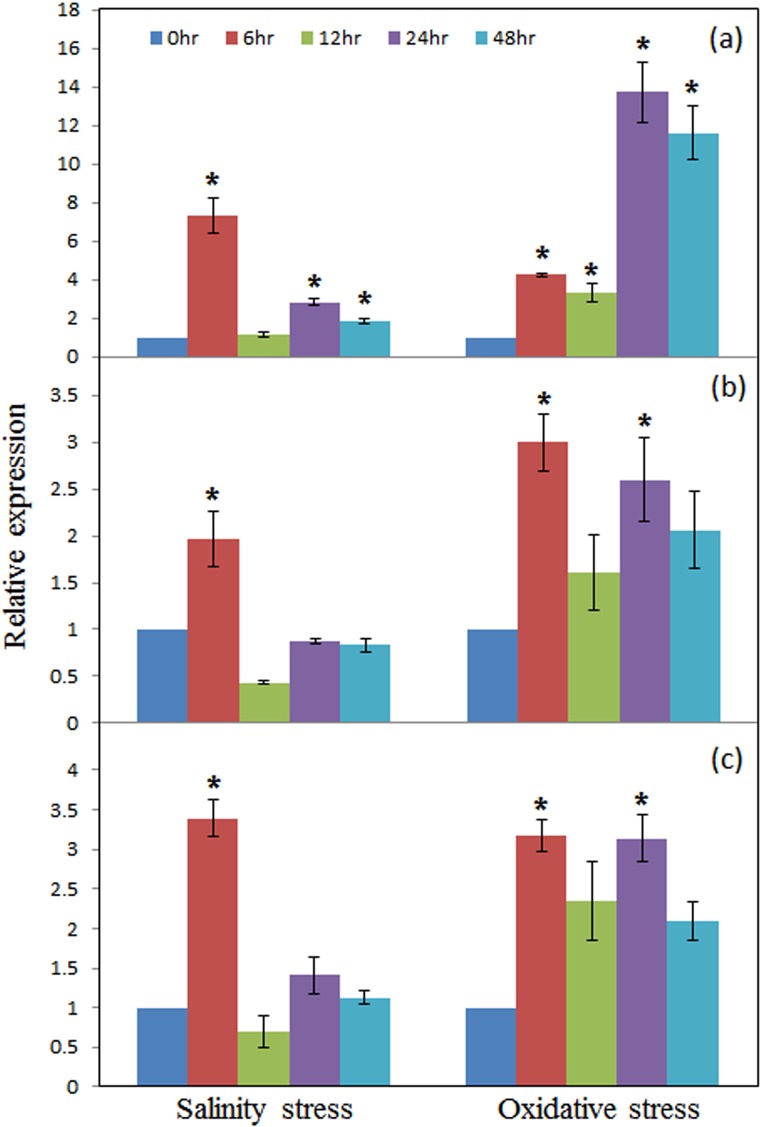
Expression analysis of selected PUFs by qRT-PCR in leaf tissue of mulberry genotype, Dudia white. Relative transcript levels of *MaUSP*-like (a), *MaPLATZ*1-like (b), and *MaRRM1*-like (c) genes under salinity and oxidative stresses. Total RNA was isolated from the leaf tissues of mulberry twigs exposed to 250mM NaCl (salinity stress) and 15μM methyl viologen (oxidative stress) at 6, 12, 24, and 48 hours after stress imposition. Transcripts were normalized to the expression of the elongation factor gene. The data shown are mean ± SE from three independent experiments. Asterisk indicates the significant difference between control and treatments at p = 0.05.

Analysis of gene expression is one of the most important approaches to highlight the functional aspect of genes. Expression pattern of the above discussed computationally annotated PUFs identified from drought specific transcriptome were studied under other abiotic stresses. Significant increase (p = 0.05) in transcript levels of PUF3 was observed under NaCl induced salt stress as well as methyl viologen induced oxidative stress (Fig 5a). The relative transcript level of PUF39 (designated as *MaPLATZ1*-like protein) was significantly up-regulated at six hours under salinity as well as oxidative stress (Fig 5b), followed by down-regulation during subsequent exposure to stress suggesting that the gene is early stress-responsive. The relative expression levels of *MaRRM1-*like genes studied under simulated salinity and oxidative stress conditions indicated significant increase at six hours after stress imposition (Fig 5c).

### Protein-protein interactions

Functional role of proteins can also be inferred by their interactions in biological networks. We derived the information from STRING database to support functional annotation using the closely related *Arabidopsis* proteins. USP-like protein from *A*. *thaliana* (AT3G17020) shares 69% identity with that of the *MaUSP-*like protein, and AT3G17020 has been shown to interact with AT3G48750 (cyclin-dependent kinase-A CDKA-1), well known for their crucial role in cell cycle regulation (S1a Fig). *MaPLATZ1*-like protein from *Arabidopsis* (AT1G21000) interacts with AT1G72830 (NF-YA3) (S1b Fig). The mulberry *MaRRM-*like protein has 65% identity with the *A*. *thaliana* gene AT4G17720. The *A*. *thaliana* protein has been reported to have direct interaction with AT5G58750 (wound responsive protein related), AT5G58690 (Phosphoinositide specific phospholipase C family protein), AT3G46060 (ATRAB8A), AT3G311730 (ATFP8), AT1G56330 (ATSAR1B), AT1G74620 (GAMMA CA2) based on experimental evidence and also with the genes like AT4G25680 and AT4G25660 (S1c Fig).

## Discussion

As a result of the revolutionary expansion in NGS technologies, a large volume of data in genome and transcriptome level has been developed, while their interpretation and generation of biological significance has been a challenge. Since, PUFs have come up as a significant portion of many genomes, we assume that their functional annotation can reveal the presence of novel candidate genes associated with growth and developmental pathways. We made an attempt to address the functional annotation of PUFs, by developing a pipeline and validating it using the drought stressed leaf transcriptome of mulberry, as an example. The pipeline involves an initial screening of the ESTs into known and unknown function against NCBI-BLAST analysis with a stringent cut off value, followed by functional annotation using computational tools.

### Pipeline of annotation

All *in silico* tools used in our pipeline (Table 3) are publicly accessible. The sequence analysis tools are efficient in identifying the presence of conserved domains, if any, in the given sequence which could be correlated to the probable function of the gene. In addition to this, the results predicted by the fold recognition tools could improve our annotation. The phylogenetic analysis as well as protein-protein interaction studies provide additional support to our function prediction. Several attempts have been made for the annotation of proteins lacking experimental supports [26][27], utilizing online computational tools in diverse organisms. Here we propose that, in addition to the *in silico* annotation, the functional relevance of annotated PUFs could be better understood, if they are taken forward to simple laboratory level experimental setup. Hence, our approach holds an improvisation over the existing ones by analyzing their expression pattern too. The expression pattern of the PUFs derived from a drought stressed library, analyzed under multiple abiotic stress conditions suggests they are stress responsive and hence may have a role in stress acclimation in mulberry.

**Table 3:** Tools used in the study.

Sl.No	Tool	Purpose	URL
I. Transcriptome assembly			
1	Newbler	*De novo* assembly	-
II. Annotation tool			
1	NCBI BLAST	Preliminary annotation of transcripts	www.ncbi.nlm.nih.gov/
III. Gene prediction			
1	FGENESH	Prediction of open reading frame	http://www.softberry.com/berry.phtml?topic=fgenesh&group=programs&subgroup=gfind
2	AUGUSTUS	Prediction of open reading frame	http://bioinf.uni-greifswald.de/augustus/submission.php
IV. Sequence analysis			
1	CDD	Identification of conserved domain	http://www.ncbi.nlm.nih.gov/Structure/cdd/wrpsb.cgi
2	Pfam	Protein family classification	http://pfam.xfam.org/search
V. Fold analysis			
1	PSIPRED	Fold recognition	http://bioinf.cs.ucl.ac.uk/psipred/
2	PHYRE2	Fold recognition	http://www.sbg.bio.ic.ac.uk/phyre2/
3	GenTHREADER	Fold recognition	http://bioinf.cs.ucl.ac.uk/psipred/
VI. Gene Ontology			
1	Gene Ontology	Annotation	http://geneontology.org/
VII. Phylogenetic analysis			
1	MEGA5		http://www.megasoftware.net/
VIII. Protein-protein interaction			
1	STRING	To find out interacting partners	http://string-db.org/
IX. Expression analysis			
1	qRT-PCR	To analyze the functional significance	-

### Annotation of PUFs using computational tools and development of PUFAS

Generally, annotation of proteins has been approached in various directions using existing computational tools. One of the simplest methods followed is relating the protein to sequence conservation and domain association. The presence of a conserved region or functional domain could be an indication of the probable functional role of the protein [28]. The PUFs that have at least one previously defined motif or domain can be called as proteins of defined features (PDFs) [28][29][4]. Fold recognition methods like threading and hybrid threading/sequence fold recognition have been widely used in assigning functions to proteins as they can often recognize even the most distant homologues. In some cases, even distantly related proteins with similar structure could be identified [30]. Hence, in addition to the sequence based prediction, we used fold recognition tools like GenTHREADER, PHYRE2 and 3DPSSM which have been extensively used in other studies [31][32][33]. We also adopted Gene Ontology (GO) annotations for identifying the function of proteins, as GO is known for giving hints to the function at various levels [3][34]. By integrating these sequence and structure based approaches we could annotate some of the PUFs from the drought specific transcriptome of mulberry (Table 1). As per our annotation, the PUFs belong to various structural/functional as well as regulatory proteins like enzymes, chaperons, signaling molecules, ribosomal proteins, TFs, etc. The PUFAS server is capable of processing the PUFs with satisfactory output in a user friendly way. Although, there are pipelines and automated servers available, which rely on well defined protein sequences [35][36], PUFAS has additional features required for NGS data analysis. The server can accept outputs from various NGS platforms, process and predict gene function.

### Analysis of expression pattern of the selected PUFs

We tried to highlight the relevance of the annotated PUFs by a random selection of three genes, one belonging to category of regulatory protein (*MaRRM1*-like), another upstream TF (*MaPLATZ1*-like) and third downstream functional protein (*MaUSP*-like) for expression analysis by qRT-PCR. From other reports, USP domain containing proteins have been up-regulated under different stress signals in plants [37]. In *Arabidopsis*, there are 44 putative homologues of USPs which are either ATP binding or non-ATP binding type [38] and the exact function of these proteins are yet to be known. PUF3 annotated as *MaUSP*-like protein, which was significantly up-regulated under multiple stresses in our study could be one of the potential candidates for imparting cellular level tolerance to abiotic stress in mulberry. PLATZ1 proteins are zinc dependent DNA binding proteins binding to AT rich regions of nucleotide sequence to bring about transcriptional repression with a possible involvement in cell cycle regulation [39]. Earlier studies report the involvement of DNA binding PLATZ1-like TF in embryo development [40] and tendril and inflorescence development [41]. In addition to the above, we propose that this PLATZ1-like gene identified from mulberry has a role in abiotic stress response, which need to be tested furthur. The PUF42 has a conserved RNA recognition motif, RRM1 which is one of the most common characteristic features of RNA binding proteins (RBPs) in plants. A wide variety of roles have been implicated for RBPs involved in abiotic stress-response in plants [42][43]. Light, salinity and abscisic acid are known to induce rapid alteration of the RBPs [41][44] as well as modulate the stress induced gene expression within minutes to hours after stress imposition [44] suggesting their involvement in early stress response. Similar to these, *MaRRM1-*like is suggested be a stress-responsive protein that might modify the stress response of the mulberry plants through mechanisms that are yet to be studied.

### Large-scale analysis of PUFs and testing of PUFAS

We selected 100 transcript sequences of known functions from *A*. *thaliana* and performed a test using the tool, PUFAS. A script, that can access the PUFAS API service, was used to perform the analysis. The results from the server were compared against the known function and identified that PUFAS could predict the same function in 93% cases (S5 File). This proves that the PUFAS API Service and the web interface can be of a great help in annotating the entire transcriptome of an organism.

### Prediction of protein-protein interactions

Protein-protein interaction is the hallmark of all living organisms [45]. More than 70% of the physically interacting proteins share similar functional annotations [46] suggesting their joint recruitment in a biological function. Hence, the functional role of proteins can also be revealed by the study of its interactions in a biological network and can be used for providing leads to functional roles of the PUFs [27]. In *Arabidopsis*, the interacting partners of USP-like protein (PUF3) i.e., CDKs have also been well related to stress perception and responses in plants there by regulating their strategies for growth, development and adaptation under biotic and abiotic cues [47][48]. Our analysis suggests that the *MaUSP-*like protein can be possibly associated with mechanisms that are related to cell cycle regulation during stress response. Recent reports suggest that NF-Y TFs have been interacting with other TF families also to influence various plant responses [49]. In this view, the *MaPLATZ1*-like protein from mulberry could also be a possible interacting partner for NF-YA3. The RRM1-like protein (PUF42) from *A*. *thaliana* has been associated with multiple pathways in growth, development and stress-response. The interaction study was attempted only to get a lead towards the probable function. However, there is a need to confirm the interactions.

## Conclusion

The strategies used in our pipeline to annotate PUFs are simple and can be used by a wide spectrum of computational as well as experimental biologists. In the present study, sequence analysis and structure-based fold predictions have been used to uncover putative biochemical functions of a few hitherto uncharacterized genes of interest. The associated biochemical functions and domains could be extended to assign possible biological functions by deriving knowledge of their homologues in other model organisms. The study of PUFs can answer some of the unanswered questions regarding the interacting partners of many proteins in many biological systems and hence can reveal additional players in the transcriptional and translational regulatory events at molecular level. The approach followed in this study can pave way for validation of many PUFs in diverse organisms. The ultimate test would be to functionally validate the selected PUFs in model or test systems by down-regulation or over expression studies. The advantage of the approach is that all the computational tools used in this study are freely accessible. The strategy demonstrated would also be adopted for annotation of PUFs at whole genome level across diverse organisms.

## Supporting Information

S1 Fig Protein-protein interaction for functional prediction of selected PUFs. a) Protein-protein interaction between AT3G17020 and CDC2. b) Protein-protein interaction between AT1G2100 and NF-YA3. c) Protein-protein interaction between AT4G17720 and AT5G58750, AT5G58690 (Phosphoinositide specific phospholipase C family protein), AT3G46060 (ATRAB8A), AT3G311730 (ATFP8), AT1G56330 (ATSAR1B), AT1G74620 (GAMMA CA2), AT4G25680 and AT4G25660.
(TIF)

S1 File Primers used for qRT-PCR experiment. (XLSX)

S2 File Annotation of the mulberry drought specific expressed sequence tags. Excel file showing the annotation of the mulberry transcripts. (XLSX)

S3 File Function prediction of PUFs in mulberry genotype, Dudia white. (XLSX)

S4 File Sample python program to access the PUFAS web service. (PY)

S5 File Testing PUFAS using 100 transcript sequences of known functions from *Arabidopsis thaliana*. (XLS)
