# Supplementary figures and images for: An Approach to Function Annotation for Proteins of Unknown Function (PUFs) in the Transcriptome of Indian Mulberry
Source: PLoS One. 2016 Mar 16;11(3):e0151323. doi: 10.1371/journal.pone.0151323 (PMC4794119; doi:10.1371/journal.pone.0151323)

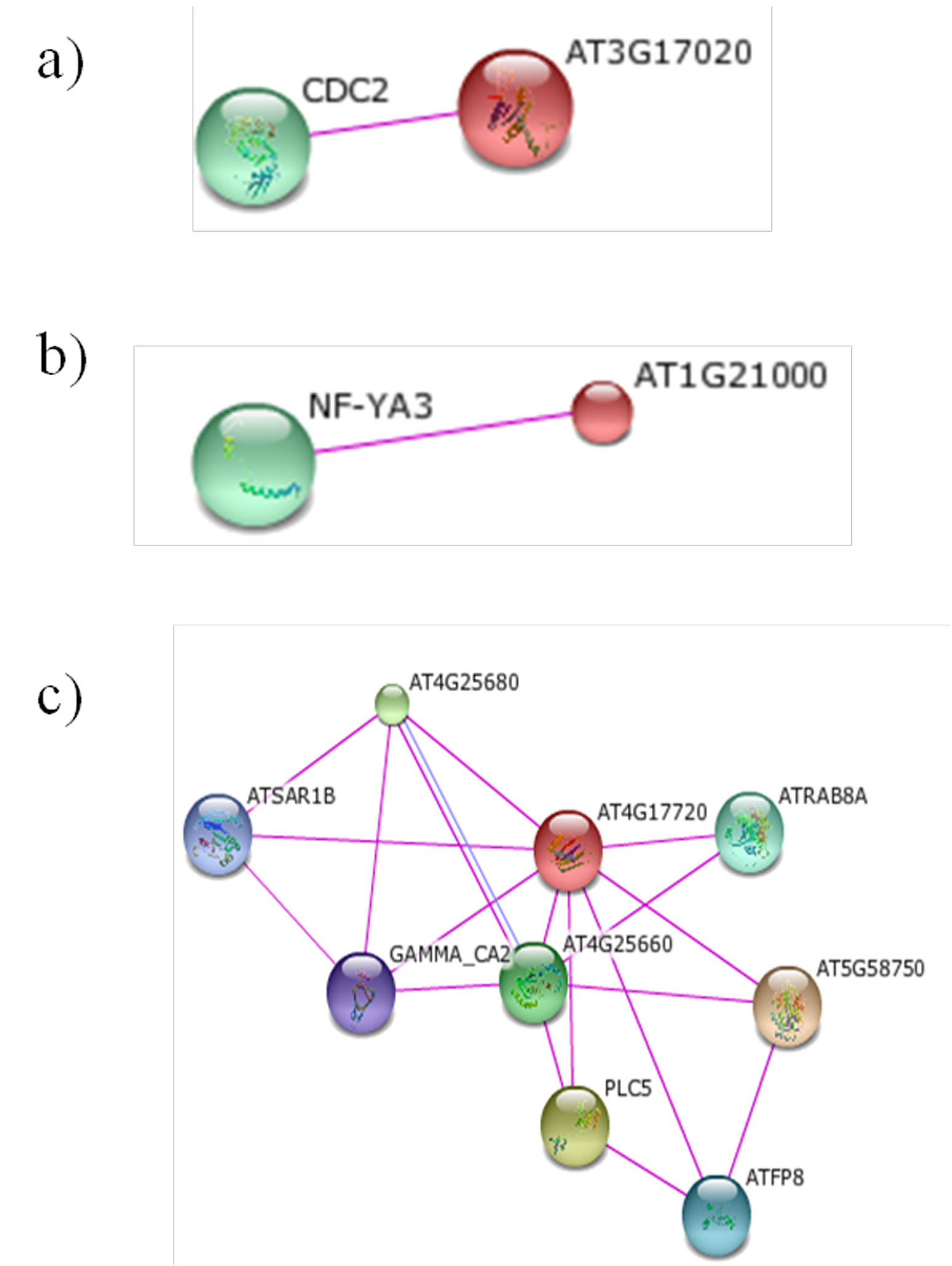

Supplement: S1 Fig — a) Protein-protein interaction between AT3G17020 and CDC2. b) Protein-protein interaction between AT1G2100 and NF-YA3. c) Protein-protein interaction between AT4G17720 and AT5G58750, AT5G58690 (Phosphoinositide specific phospholipase C family protein), AT3G46060 (ATRAB8A), AT3G311730 (ATFP8), AT1G56330 (ATSAR1B), AT1G74620 (GAMMA CA2), AT4G25680 and AT4G25660. (TIF) [file pone.0151323.s001.tif]
